# Do Grape- and Wine-Derived (Poly)Phenols Contribute to the Hallmarks of Healthy Brain Ageing?

**DOI:** 10.3390/nu18193144

**Published:** 2026-09-24

**Authors:** Ayman Ben Mohamed, David Vauzour

**Affiliations:** Norwich Medical School, University of East Anglia, Norwich NR4 7TJ, UK; aymoben2@gmail.com

**Keywords:** grape- and wine-derived (poly)phenols, grape-derived extracts, grape seed extract, grape pomace, proanthocyanidins, resveratrol, phenolic metabolites, bioavailability, gut microbiota, gut-brain axis, cognitive ageing, mild cognitive impairment, neuroinflammation

## Abstract

Population ageing is associated with a rising burden of cognitive decline, dementia and late-life affective disorders. Grape- and wine-derived (poly)phenols represent a chemically diverse group of bioactives whose biological relevance is shaped by structural complexity, the intervention matrix, host conjugation and gut microbial metabolism. Rather than acting as simple antioxidants, these compounds and their derived metabolites may influence interrelated pathways implicated in brain ageing, including neuroinflammation, endothelial function, synaptic plasticity, oxidative stress and gut-brain axis signalling. The gut microbiota is central to this framework as it converts poorly absorbed parent compounds into smaller phenolic metabolites and is modulated by (poly)phenol exposure. This review summarises the chemical and bioavailability features that determine intestinal and colonic availability, evaluates preclinical evidence concerning the neuroprotective potential of grape- and wine-derived (poly)phenols, and integrates clinical evidence from studies of cognitive ageing, mild cognitive impairment and Alzheimer’s disease. Current evidence supports biological plausibility and justifies further investigation, but clinical findings remain heterogeneous. Future studies should prioritise chemically standardised preparations, metabolomic profiling, functional microbiome readouts, and integrated cognitive, vascular and inflammatory endpoints.

## 1. Introduction

The progressive ageing of the global population represents a major and escalating public health challenge. Although increased life expectancy reflects important medical and societal advances, it is accompanied by a substantial rise in the prevalence of neurodegenerative and neuropsychiatric disorders. Currently, more than 50 million individuals worldwide are living with dementia, with projections reaching 152 million by 2050 [1]. In parallel, a large proportion of older adults experience depression, anxiety, and other mood disorders, which frequently coexist with cognitive decline and exacerbate functional impairment and loss of independence [2,3].

Brain ageing is driven by interacting biological processes, including oxidative stress, chronic inflammation, vascular dysfunction, and blood–brain barrier impairment [4], which collectively increase neuronal vulnerability in memory-related regions such as the hippocampus and prefrontal cortex. These central nervous system changes are influenced by systemic factors, including metabolic dysregulation, immune signalling, and gut microbiota composition, highlighting the role of the gut-brain axis in brain ageing [5,6].

In this context, nutritional strategies have emerged as promising approaches to counteract age-related cognitive and mental decline [7]. Among dietary bioactives, (poly)phenols have received considerable attention due to their wide distribution in plant-based foods and their capacity to modulate multiple biological systems. These compounds may influence neuronal signalling, vascular function, and gut microbiota activity, supporting overall brain health through multiple converging mechanisms [8,9,10]. The Mediterranean diet, characterised by a high intake of fruits, vegetables, legumes, nuts, and olive oil, together with moderate consumption of red wine, has been consistently associated with better cognitive outcomes and reduced risk of neurodegenerative diseases [11]. Within this framework, grape- and wine-derived (poly)phenols, including flavonoids and stilbenes such as resveratrol, have attracted particular interest. These (poly)phenols are relevant because they may support vascular and neuronal resilience and interact with the gut microbiota to generate bioactive metabolites [12,13]. Epidemiological data suggest that moderate red wine consumption is associated with reduced cognitive decline, although residual confounding persists [14,15]. In addition, grape- and wine-derived (poly)phenols may limit the production of deleterious metabolites such as trimethylamine *N*-oxide (TMAO), thereby contributing to a more favourable metabolic environment for brain ageing [16]. Collectively, current evidence suggests that grape- and wine-derived (poly)phenols may act as multi-system bioactive compounds influencing metabolic, microbial, vascular, and neuronal pathways, rather than functioning solely as antioxidants [17].

This review critically evaluates the potential contribution of grape- and wine-derived (poly)phenols to cognitive ageing and mental health within an exposure-specific translational framework. The term “grape- and wine-derived (poly)phenols” encompasses phenolic compounds delivered through alcoholic and dealcoholised wines, grape-derived food matrices, grape seed and pomace preparations, mixed grape-based extracts and isolated compounds such as resveratrol. Throughout the review, evidence is attributed to the specific preparation studied, and findings from isolated compounds or grape-derived extracts are not assumed to represent the effects of alcoholic or dealcoholised wine. Rather than treating these compositionally distinct exposures as a single intervention class or interpreting antioxidant capacity as a sufficient mechanism, we consider their phenolic profiles, matrices, doses, bioavailability, pharmacokinetics and ethanol content separately. We integrate evidence concerning the biological processes underlying cognitive decline and mood disorders with preclinical and clinical findings relating these interventions to cognitive, psychological, vascular and neuroimaging outcomes. Particular attention is given to oxidative stress, inflammation, vascular function, synaptic plasticity and gut-brain axis signalling, including the dual contribution of microbial metabolites as potential risk or protective factors in brain ageing. A central feature of this review is its assessment of whether changes in the gut microbiota or circulating metabolites are accompanied by measurable cognitive, vascular or neuroimaging effects, thereby distinguishing mechanistic plausibility from demonstrated clinical effectiveness.

## 2. Methods and Literature Selection

This narrative review was informed by structured searches of PubMed/MEDLINE and Scopus. Searches combined terms relating to exposure (“grape”, “wine”, “red wine”, “dealcoholised wine”, “grape seed”, “grape pomace”, “proanthocyanidin”, “anthocyanin”, “resveratrol” and “polyphenol”) with terms relating to the population and outcomes (“ageing”, “older adults”, “mild cognitive impairment”, “Alzheimer’s disease”, “cognition”, “memory”, “mood”, “brain”, “cerebral blood flow”, “gut microbiota”, “microbial metabolite”, “short-chain fatty acid”, “TMAO”, “bioavailability” and “clinical trial”). Eligible evidence comprised English-language peer-reviewed human intervention studies, observational studies, systematic reviews and meta-analyses, together with mechanistic animal studies directly relevant to grape- or wine-derived (poly)phenols and brain ageing pathways. For the clinical synthesis, priority was given to randomised or controlled interventions reporting cognitive, mood, cerebrovascular, neuroimaging, inflammatory, metabolomic or microbiota outcomes. Studies were excluded when the exposure was unrelated to grape- or wine-derived (poly)phenols; the outcome had no clear relevance to ageing, cognition, mental health or the gut-brain axis; essential intervention details were unavailable; or the report was a conference abstract or protocol without outcome data. Protocols and interim reports were retained only to identify emerging directions and were not treated as evidence of efficacy.

## 3. Ageing, Associated Factors, Cognitive Decline and Mental Health

Age-related cognitive decline is characterised by a progressive and heterogeneous deterioration of specific cognitive domains, notably episodic memory, processing speed, and attentional capacity. In contrast, abilities such as vocabulary and accumulated knowledge are relatively preserved. When these alterations exceed normative ageing trajectories, they are classified as mild cognitive impairment (MCI), a condition affecting approximately 15% of individuals over 65 years of age [18]. Importantly, MCI represents a transitional state associated with an elevated risk of progression to Alzheimer’s disease (AD) and other dementias, although trajectories remain variable and are influenced by genetic, metabolic, and environmental factors [19,20].

From a neurobiological perspective, ageing is associated with progressive structural and functional brain alterations. These include hippocampal and entorhinal cortex atrophy, reduced white matter integrity, and disrupted large-scale network connectivity, all of which contribute to impaired cognitive performance [21]. These changes are further exacerbated by the accumulation of pathological hallmarks such as amyloid-β deposition and tau pathology, alongside mitochondrial dysfunction, impaired proteostasis, and chronic neuroinflammation [22,23]. Importantly, these processes are not independent and rather interact within a complex network of cellular stress responses that collectively drive neuronal dysfunction and loss.

Beyond cognitive decline, ageing is also associated with increased vulnerability to mental health disorders, particularly late-life depression and anxiety. These conditions frequently co-occur with cognitive impairment and may act both as risk factors and prodromal manifestations of neurodegenerative disease [24]. Dysregulation of the hypothalamic-pituitary-adrenal (HPA) axis, alterations in serotonergic and dopaminergic neurotransmission, and chronic low-grade inflammation contribute to the development of affective symptoms in older adults [25,26]. Increasing evidence further suggests that these neuropsychiatric conditions share common biological features with neurodegeneration, including immune activation, vascular dysfunction, and metabolic imbalance.

The gut microbiota has emerged as a central regulator of these processes. Ageing is associated with a decline in microbial diversity, loss of beneficial taxa, and expansion of opportunistic pathobionts, resulting in an altered metabolic output and reduced resilience of the intestinal ecosystem [27,28]. These changes are accompanied by decreased production of protective metabolites such as short-chain fatty acids (SCFAs) and increased generation of potentially harmful compounds, including indoles, trimethylamine *N*-oxide (TMAO), and secondary bile acids [29,30,31,32]. Such alterations may promote systemic inflammation, increase intestinal permeability, and compromise blood-brain barrier integrity, thereby facilitating the transmission of inflammatory and metabolic signals to the central nervous system. This gut-brain axis dysregulation is increasingly recognised as a key contributor to both cognitive decline and mood disorders in ageing populations [33] (Figure 1).

Taken together, the convergence of cognitive decline and mental health deterioration highlights the multifactorial nature of brain ageing and underscores the limitations of single-target pharmacological approaches. In this context, preventive and integrative strategies targeting multiple interconnected pathways are required. Nutritional interventions have attracted particular interest due to their capacity to simultaneously modulate metabolic, inflammatory, and microbial processes. Grape- and wine-derived (poly)phenols, owing to their widespread dietary presence and their ability to influence both host physiology and gut microbiota composition, represent promising candidates in this regard [8,10,34]. By targeting key mechanisms underlying neurodegeneration and neuropsychiatric vulnerability, they may contribute to preserving cognitive function and emotional well-being during ageing.

## 4. Chemical Composition and Bioavailability of Grape- and Wine-Derived (Poly)Phenols

Grape- and wine-derived (poly)phenols comprise a structurally diverse group of flavonoid and non-flavonoid compounds originating from grape- and winemaking-related transformations. The principal flavonoid classes include flavan-3-ols, flavonols, anthocyanins and condensed tannins, whereas non-flavonoids encompass hydroxycinnamic acids, hydroxybenzoic acids, stilbenes and related phenolic derivatives [35,36]. Although traditionally associated with the sensory properties of wine, these compounds are increasingly recognised for their potential to modulate pathways relevant to brain ageing, including oxidative stress, neuroinflammation and cerebrovascular function [10,36]. Their abundance and composition vary according to grape cultivar, ripeness and processing conditions such as maceration, fermentation and storage [36,37].

Flavan-3-ols vary markedly in their degree of polymerisation, which influences intestinal absorption, microbial metabolism and potential biological activity [37,38]. Their degree of polymerisation strongly influences bioavailability and, consequently, their potential impact on neurobiological processes. While monomeric and low-molecular-weight forms may be absorbed in the small intestine, highly polymerised compounds reach the colon, where they undergo extensive microbial catabolism [39,40]. This process generates smaller metabolites, including phenyl-γ-valerolactones and phenolic acids, that can enter the systemic circulation. Experimental studies suggest that selected low-molecular-weight phenolic metabolites may cross or interact with the blood-brain barrier and influence neuroinflammatory signalling, neuronal resilience and synaptic plasticity [41,42,43,44]. However, direct evidence demonstrating the identity, concentration and regional distribution of dietary (poly)phenols and their metabolites in human brain tissue remains lacking, although their detection in human cerebrospinal fluid supports their central bioavailability [12,45].

Anthocyanins, which primarily occur as glycosides of malvidin, delphinidin, petunidin, peonidin, and cyanidin, are abundant in grapes and red wine and are highly susceptible to changes in physicochemical conditions and metabolic transformations [36,38]. Despite low bioavailability of the parent compounds due to instability and rapid metabolism, their downstream metabolites may represent key mediators of biological activity. These metabolites have been implicated in mechanisms relevant to neurodegeneration, including modulation of oxidative stress responses and maintenance of blood-brain barrier integrity, suggesting that circulating phenolic derivatives, rather than intact anthocyanins, are likely to drive neuroprotective effects [46,47,48]. Wine also contains anthocyanin-derived pyranoanthocyanins, including vitisins [49,50] and pinotins [51], which are formed through the transformation of grape-derived anthocyanins during fermentation and ageing. Although generally minor constituents, their abundance depends on winemaking conditions and interactions with other phenolics, including grape seed tannins.

Flavonols (e.g., quercetin and kaempferol) and stilbenes, particularly resveratrol, represent smaller but functionally relevant components of grape- and wine-derived (poly)phenols [36,37]. These compounds and their metabolites have been associated with modulation of mitochondrial function, inflammatory signalling and cellular stress responses, all of which are central to the pathophysiology of age-related cognitive decline and neurodegenerative diseases [52,53]. However, rapid metabolism limits systemic exposure to parent compounds, reinforcing the importance of conjugated and microbiota-derived metabolites as putative bioactive species [8].

The biological effects of grape- and wine-derived (poly)phenols are further shaped by matrix interactions with fibre, proteins and cell wall components, which influence release, digestion and microbial accessibility [54,55]. This is particularly relevant in complex matrices such as grape-derived products, where poorly digestible components promote delivery of (poly)phenols to the colon, thereby enhancing microbiota-driven metabolism. Given the links between the gut microbiota and brain ageing, this colonic phase of metabolism may be important for generating systemically available metabolites with potential relevance to gut-brain signalling [44,56]. Bioavailability should therefore be considered a dynamic, multi-step process encompassing release from the food matrix, intestinal absorption, host metabolism, microbial transformation and systemic distribution. Because a substantial proportion of grape- and wine-derived (poly)phenols reaches the colon, microbiota-dependent conversion into lower molecular weight metabolites is a key determinant of their bioactivity [40,57]. These circulating metabolites differ markedly from the parent compounds and are more likely to cross biological barriers and exert effects on neural and vascular tissues, making them more relevant markers of exposure in the context of brain ageing [41,58,59].

Inter-individual variability in gut microbiota composition and function is likely to also critically influence responsiveness to grape- and wine-derived (poly)phenols. Age-related declines in microbial diversity and metabolic capacity may reduce the generation of neuroactive phenolic metabolites, whereas microbiota profiles enriched in specific catabolic pathways may enhance their production and systemic availability [27,29,60,61]. This variability may contribute to differences in cognitive and neurobiological outcomes observed in grape- and wine-derived (poly)phenol intervention studies. Future studies should therefore incorporate assessments of microbiota composition and phenolic metabolite profiles to better define responder phenotypes and support personalised strategies targeting neurodegeneration and healthy brain ageing.

## 5. Impact of Grape- and Wine-Derived (Poly)Phenols on Brain Health

Preclinical studies provide strong mechanistic support for the role of grape- and wine-derived (poly)phenols in modulating processes central to brain ageing and neurodegeneration. These compounds have been reported to regulate endogenous antioxidant defences and inflammatory pathways, including redox-sensitive signalling, NF-κB-mediated responses and cytokine production [8,10,44,62,63,64], while also influencing longevity-associated pathways such as SIRT1 activation, mitochondrial function, neurotrophic signalling and synaptic plasticity [53,65,66,67]. These mechanisms align closely with the pathophysiological processes underlying age-related cognitive decline, where oxidative stress, mitochondrial dysfunction and chronic neuroinflammation converge.

More recent studies extend this framework, demonstrating effects of grape- and wine-derived (poly)phenols on core hallmarks of neurodegeneration, including amyloid-related toxicity, autophagy and dopaminergic vulnerability [68,69,70,71,72,73,74]. Grape- and wine-derived (poly)phenols and their metabolites modulate protein aggregation, enhance cellular stress responses and regulate neurotrophic signalling, thereby supporting neuronal resilience during ageing. These effects are partly mediated by microbiota-derived metabolites acting along the gut-brain axis to regulate neuroinflammatory and oxidative pathways [40,41,75], consistent with emerging evidence that (poly)phenols target multiple interconnected hallmarks of neurodegeneration, including impaired proteostasis, mitochondrial dysfunction and defective autophagy [70,74].

Experimental data also support a role for grape seed procyanidins in proteostasis and cellular quality control. In a transgenic *Caenorhabditis elegans* model expressing human amyloid-β, a grape seed (poly)phenol extract attenuated amyloid-induced paralysis, extended lifespan and modulated genes involved in autophagy and protein homeostasis [69]. Although this model does not capture the complexity of mammalian cognition, it provides mechanistic evidence linking grape-derived phenolics to aggregation-related toxicity relevant to Alzheimer’s disease. Furthermore, (poly)phenol-rich grape and blueberry extracts prevent age-related cognitive decline in mice, enhancing hippocampal neurogenesis and increasing neurotrophic factor expression [76]. Similarly, dietary (poly)phenols restored spatial learning and synaptic plasticity-associated signalling pathways, including CaMKII and neurotrophic signalling, in middle-aged animals [77]. The grape seed extract further promoted hippocampal neurogenesis and synaptogenesis while improving behavioural performance in aged mice [78]. More recent work reported that epicatehin, a grape-derived (poly)phenol, improved cognitive outcomes while restoring antioxidant enzyme activity and reducing oxidative damage in the brain, directly linking redox regulation to behavioural performance [79]. In parallel, grape seed procyanidins preserved neurotrophic and muscarinic signalling pathways in aged animals, supporting their role in maintaining synaptic and neuromuscular function during ageing [80]. Collectively, these findings are consistent with broader evidence that grape- and wine-derived (poly)phenols enhance neuronal survival and synaptic plasticity through coordinated regulation of oxidative stress and neuroinflammatory pathways [44].

Grape-derived (poly)phenols have also been investigated in models of dopaminergic neurodegeneration. In the MPTP mouse model, the grape pomace extract preserved dopaminergic neuronal markers and attenuated neurotoxic damage [81]. Complementary evidence shows that grape seed procyanidins mitigate oxidative-stress-induced cognitive impairment, restore hippocampal antioxidant activity and modulate signalling pathways such as NR2B/CREB [82]. More broadly, experimental models demonstrate that grape- and wine-derived (poly)phenol-related compounds suppress key inflammatory mediators, including NF-κB, TNF-α and IL-1β, supporting their role in targeting shared mechanisms across Alzheimer’s and Parkinson’s disease pathology [41]. Importantly, preclinical studies highlight the influence of extract composition and formulation on efficacy. Variability in phenolic profiles can lead to divergent neurobiological outcomes, emphasising that grape- and wine-derived (poly)phenols should be considered composition-specific interventions rather than interchangeable mixtures [83]. Finally, additional mechanistic evidence supports the involvement of grape-derived (poly)phenols in neurotransmission and neuromodulation. Grape seed extracts were reported to modulate GABAergic, monoaminergic and cholinergic systems and to protect neuronal cells under oxidative stress [84], while also influencing neurotrophic and muscarinic signalling pathways during ageing [80]. These findings extend the mechanistic scope of grape- and wine-derived (poly)phenols beyond antioxidant activity, highlighting their capacity to modulate neurotransmission, neurotrophic signalling and cellular resilience in the ageing brain (Figure 2).

Human clinical evidence remains less extensive and more heterogeneous than the preclinical literature. Published clinical studies investigating brain function have employed a range of interventions, including grape-derived extracts, grape seed and pomace extracts, resveratrol supplements, and mixed grape–berry (poly)phenol preparations [85,86,87,88]. For example, a randomised, double-blind, placebo-controlled study tested a (poly)phenol-rich grape and blueberry extract (Memophenol^®^) in 143 adults aged 60–80 years with mild cognitive impairment. Participants received 150 mg twice daily for six months. Compared with the placebo, the intervention was associated with greater improvements in speed of information processing, visuospatial learning and the BRIEF-A global score, while other cognitive and self-report outcomes did not differ significantly [89]. These findings suggest potential domain-specific benefits but also indicate that effects are not uniform across cognitive measures. More recently, a double-blind, placebo-controlled trial with a standardised grape extract (250 mg/day Cognigrape^®^ for 12 weeks) in 96 healthy older adults reported statistically significant improvements across several cognitive domains, including immediate and delayed memory, visuospatial abilities, language and attention, with some changes reported after 14 days and continued improvement after 84 days [90]. Although this study used a healthy older population rather than individuals with established impairment, it supports further evaluation of grape- and wine-derived (poly)phenolic preparations in cognitive ageing. Conversely, a randomised, double-blind, placebo-controlled trial evaluated grape seed procyanidin extracts (320 mg/day for six months) in adults 60 years or older with mild cognitive impairment; it was reported that MoCA scores increased in both groups, but the between-group change did not differ significantly [91] (Table 1). The divergent clinical findings are likely to reflect several interacting factors rather than a simple dose-response relationship. The null between-group MoCA result with grape seed procyanidins may relate to improvement in both intervention and placebo groups, limited sensitivity of a global screening instrument to domain-specific change, baseline cognitive status, extract composition or insufficient exposure to relevant circulating metabolites. By contrast, positive studies used different preparations, populations and multidomain cognitive batteries and sometimes detected changes in selected rather than global outcomes. A systematic review of randomised controlled trials of grape interventions and cognitive or mental performance also supported cautious interpretation. Chronic interventions produced improvements in some cognitive domains, whereas acute interventions showed no consistent effects on memory, although selected improvements in reaction time were reported [87]. Differences in study design, dosage and outcome tests more likely hindered comparison across studies.

Resveratrol trials provide complementary but compositionally distinct evidence and should not be extrapolated to wine or complex grape matrices. In healthy overweight older adults, a 26-week intervention with resveratrol (200 mg/day) improved word retention and increased hippocampal functional connectivity, accompanied by changes in glucose-related measures [12]. In mild-to-moderate Alzheimer’s disease, a 52-week phase 2 randomised, double-blind, placebo-controlled trial involving 119 participants found that resveratrol and its major metabolites were measurable in plasma and cerebrospinal fluid. The intervention was reported to be safe and well-tolerated and altered some biomarker trajectories; however, the clinical significance of these changes remained uncertain [92]. More recently, a 12-week randomised, double-blind, placebo-controlled trial evaluated 500 mg/day resveratrol in 99 adults aged 35–60 years with overweight or obesity, integrating cognitive testing with gut microbiota, urinary metabolomic, inflammatory and oxidative measurements. Baseline differences in cognitive outcomes in the placebo group complicated interpretation, and no significant within- or between-group differences were observed for microbiome, urinary metabolome, adiposity, inflammatory or oxidative markers. The study therefore did not provide definitive evidence of cognitive benefit or microbiota-mediated effects [93]. Resveratrol trials introduce further differences in absorption, metabolism and target engagement because an isolated stilbene is not equivalent to a procyanidin-rich extract or mixed grape-berry matrix. Population health status, intervention duration, background diet, microbiota-dependent metabolite production, chemical standardisation, multiplicity of cognitive endpoints and correction for multiple testing may therefore all contribute to apparently inconsistent results. These contrasts argue against pooling the interventions conceptually and support preparation-specific interpretation with emphasis on prespecified primary outcomes and effect sizes (Table 1).

Overall, the clinical evidence supports plausibility but does not yet have a definitive translation. Positive findings are most consistent for selected cognitive domains, whereas global cognitive effects are less consistent. More broadly, clinical translation requires chemically standardised preparations, detailed metabolite profiling and integration of cognitive outcomes with vascular, inflammatory and microbiota-derived biomarkers (Figure 2).

## 6. Gut Microbiota Modulation by Grape- and Wine-Derived (Poly)Phenols: Linking Composition, Metabolism and Function

The gut microbiota represents both a biological target and a metabolic partner of grape- and wine-derived (poly)phenols. This relationship is closely linked to the chemical and bioavailability features described above. Many grape- and wine-derived phenolics, particularly highly polymerised proanthocyanidins, anthocyanins, flavan-3-ols and matrix-associated phenolics, are only partially absorbed in the upper gastrointestinal tract. As a result, a substantial fraction reaches the colon, where it becomes available for microbial biotransformation [13,40,54,57,94,95]. Through this process, intestinal microorganisms convert poorly absorbed parent compounds into smaller phenolic acids, valerolactone-related metabolites and other aromatic catabolites that may act locally within the gut or enter the circulation and exert systemic effects [40,57,96,97].

This reciprocal interaction means that microbiota modulation by grape- and wine-derived (poly)phenols should not be interpreted solely as a change in microbial taxonomy. Rather, it reflects a combination of selective microbial utilisation of phenolic substrates, antimicrobial or growth-modulating effects of parent compounds and metabolites and altered microbial metabolic output. These functional consequences may be more biologically relevant than shifts in relative abundance alone. Evidence of human intervention supports this concept. In a controlled crossover study, moderate intake of red wine or dealcoholised red wine (poly)phenols for 20 days altered selected faecal microbial groups, including *Enterococcus*, *Prevotella*, *Bacteroides*, *Bifidobacterium*, *Bacteroides uniformis*, *Eggerthella lenta* and the *Blautia coccoides*-*Eubacterium rectale* group [13]. Although this study was small, short-term and not designed to evaluate cognitive or brain outcomes, it provides important mechanistic evidence that grape- and wine-derived (poly)phenolic compounds can influence the human gut microbial ecosystem.

Grape pomace is particularly relevant in this context because it combines phenolic compounds with dietary fibre, oligosaccharides and cell-wall material, thereby providing both phenolic and fermentable substrates for the gut microbiota. Recent reviews emphasise that grape pomace contains bioactive phenolics and oligosaccharides that can reach the colon and interact with microbial communities, with in vitro evidence suggesting stimulation of selected commensal bacteria and inhibition of some potentially pathogenic bacteria [54,95]. However, human data indicate a more nuanced picture. In a randomised crossover trial involving subjects at cardiometabolic risk, grape pomace supplementation did not significantly modify the major microbial groups measured in the full cohort, although subgroup-specific effects were observed [94]. This suggests that baseline metabolic phenotype, microbial ecology, analytical resolution and responder status may strongly influence whether microbiota changes are detectable.

The biological significance of microbiota modulation by grape- and wine-derived (poly)phenols is therefore likely to depend less on taxonomy alone and more on microbial metabolite production. Several metabolite classes provide plausible links between grape- and wine-derived (poly)phenol composition and biological function. Phenolic acids and valerolactone-related metabolites represent one major route through which poorly absorbed flavan-3-ols and proanthocyanidins may acquire systemic relevance [40,96,97]. These metabolites have been discussed in relation to anti-inflammatory, vascular and neuroprotective mechanisms, although direct evidence linking specific grape- and wine-derived microbial metabolites to improved cognitive outcomes in humans remains limited [40,96,98]. SCFAs provide a second functional layer. Although SCFAs are primarily generated through microbial fermentation of fermentable carbohydrates, grape- and wine-derived matrices that include fibre, oligosaccharides or cell-wall material may influence saccharolytic fermentation and SCFA availability [54,95,98]. SCFAs are relevant to the microbiota-gut-brain axis because they have been implicated in immune regulation, epithelial barrier integrity, endocrine signalling, vagal communication, blood-brain barrier function and neuroinflammatory tone [99,100]. Butyrate is of particular mechanistic interest because of its roles in epithelial integrity, immune regulation and epigenetic control of gene expression [101]. However, evidence that grape- and wine-derived (poly)phenols improve cognition specifically through SCFA-mediated signalling remains limited. Consequently, this pathway should be considered a plausible mechanistic hypothesis rather than an established clinical mechanism [102,103].

Microbial metabolites associated with cardiometabolic and neuroinflammatory risk pathways are also relevant. TMAO, secondary bile acids and tryptophan-derived metabolites have been implicated in vascular dysfunction, epithelial and blood-brain barrier integrity, inflammatory tone, neurotransmitter balance and neuroimmune signalling [31]. Grape pomace polyphenolic formulations have been reported to reduce circulating TMAO in preliminary human studies, including randomised or clinical supplementation designs using a grape pomace extract formulation [16,104,105]. These studies were not cognition trials and should not be interpreted as evidence of cognitive benefit. Nevertheless, they are relevant to cognitive ageing because cardiometabolic dysfunction, vascular risk and systemic inflammation are recognised contributors to late-life cognitive decline. TMAO should therefore be considered a candidate mechanistic biomarker linking microbial metabolism, vascular risk and brain-relevant ageing pathways [106], rather than a proven mediator of grape- and wine-derived (poly)phenol effects on cognition.

Cognitive ageing and late-life mental health disorders share several biological substrates, including inflammation, HPA-axis dysregulation, altered monoaminergic neurotransmission, oxidative stress and tryptophan-kynurenine pathway imbalance [5,107]. Grape- and wine-derived (poly)phenols may be relevant to these processes through direct and microbiota-mediated mechanisms. In vitro evidence indicates that standardised grape seed extract can modulate enzymes and receptor systems relevant to mood and cognition, including GABA transaminase, monoamine oxidase A, acetylcholinesterase and GABA-A receptor binding [84]. However, clinical evidence remains preliminary. Future studies should include validated affective outcomes, sleep measures and biological markers of inflammation and tryptophan metabolism to determine whether microbiota-mediated metabolism of grape- and wine-derived (poly)phenols contributes to emotional resilience in ageing.

## 7. Translational Challenges and Future Directions

The available evidence has several important limitations that must be addressed before grape- and wine-derived (poly)phenols can inform evidence-based recommendations concerning cognitive or mental health. Clinical interventions employ heterogeneous and compositionally distinct preparations, including grape seed and pomace extracts, mixed grape-berry formulations and isolated resveratrol, which differ substantially in phenolic profile, matrix, dose, bioavailability and pharmacokinetics and should therefore not be treated as equivalent exposures [52,55,84,85]. Many trials are small, short in duration and insufficiently powered to detect modest cognitive effects, while inconsistent chemical characterisation, particularly of individual phenolics, proanthocyanidin degree of polymerisation, metabolite exposure and batch-to-batch reproducibility, limits comparisons across studies. The use of multiple cognitive tests and reporting of effects across different cognitive domains further increases the possibility of selective or chance findings when primary outcomes and multiplicity adjustments are unclear. Responses may also be modified by baseline microbiota composition, metabolic status, dietary background and individual capacity to generate circulating phenolic metabolites [40,94,95]. Mechanistic interpretation is additionally constrained by the frequent separation of microbiota, metabolite and brain outcomes. Indeed, changes in microbial relative abundance, SCFAs, TMAO or circulating phenolic metabolites do not demonstrate that these alterations mediate cognitive benefits. Few human studies integrate repeated measures of chemically verified exposure, microbiome function, metabolomics, vascular or neuroimaging endpoints and cognition within the same design, and formal mediation analyses remain rare. Preclinical studies often use doses or concentrations exceeding realistic dietary exposure, while observational studies remain vulnerable to residual confounding and cannot establish causality. Future studies should therefore prioritise functional microbiome and metabolomic readouts over taxonomic profiling alone and test mediation rather than infer it from parallel changes. They should also report total phenolic content, individual compound profiles, proanthocyanidin degree of polymerisation, anthocyanin and phenolic-acid composition, stilbene and relevant matrix characteristics. Adequately powered, longer-duration randomised trials should prespecify primary cognitive outcomes, apply appropriate multiplicity control, report effect sizes and confidence intervals, and integrate mood, vascular, inflammatory, neuroimaging and dietary measures where feasible. Predefined responder analyses based on baseline microbial and metabolic phenotypes may be particularly informative in older adults, recognising that individuals can differ markedly in their generation of phenolic acids, valerolactone-related metabolites, SCFAs and other signalling molecules from the same preparation [108]. Accordingly, the current evidence supports biological plausibility and hypothesis generation but not established clinical effectiveness or recommendations to consume alcoholic wine.

## 8. Conclusions

Brain ageing and late-life mental health are influenced by interacting inflammatory, vascular, metabolic, proteostatic and microbiota-related processes. Grape- and wine-derived (poly)phenols and their metabolites can engage several of these pathways in experimental systems, providing a biologically plausible basis for further investigation. However, the human evidence does not establish clinical effectiveness. Findings vary by preparation, matrix, dose, duration, population and cognitive outcome, and direct evidence linking microbiota-related changes or circulating metabolites to improved brain function remains limited. Grape- and wine-derived extracts, mixed preparations and isolated resveratrol should therefore be interpreted as distinct exposures. Observational associations between moderate wine intake and cognitive outcomes should not be interpreted as causal evidence or as support for initiating or increasing alcohol consumption. Findings obtained with dealcoholised products, extracts or supplements primarily inform the biological effects of phenolic constituents and cannot be extrapolated to alcoholic wine. Where alcoholic wine is studied, potential phenolic effects must be distinguished from those of ethanol using appropriately matched comparators. Future studies require chemically standardised interventions, adequate power and duration, prespecified cognitive outcomes, robust control of multiplicity, detailed exposure metabolomics and integrated microbiome, vascular, inflammatory and neuroimaging measures. At present, the most defensible conclusion is that grape- and wine-derived (poly)phenols provide mechanistic and biological plausibility, but their capacity to preserve cognition or mental health during ageing remains to be established in rigorous human trials. Any potential benefit should be considered within broader dietary and lifestyle strategies and should not be interpreted as evidence supporting the initiation or increased consumption of alcoholic wine.

## Figures and Tables

**Figure 1:**
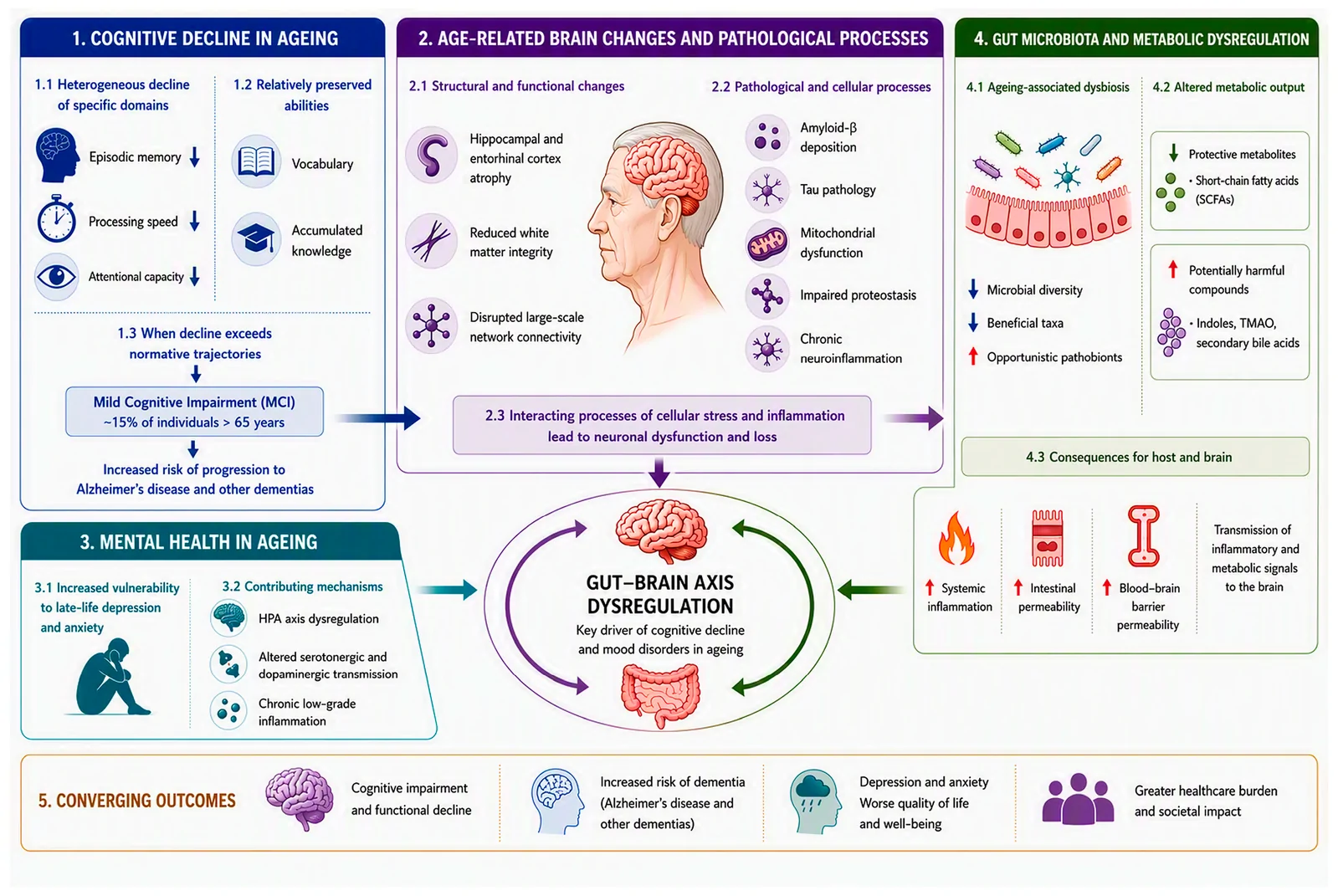
Ageing-related gut-brain axis dysregulation and brain health decline. Ageing is associated with selective cognitive decline, increased risk of mild cognitive impairment and dementia, structural and pathological brain changes, and greater vulnerability to late-life depression and anxiety. These processes interact with age-related gut microbiota dysbiosis, altered microbial metabolite production, systemic inflammation, increased intestinal permeability and impaired blood-brain barrier integrity. Together, these interconnected mechanisms contribute to gut-brain axis dysregulation, supporting cognitive impairment, neuropsychiatric vulnerability and broader functional decline in later life.

**Figure 2:**
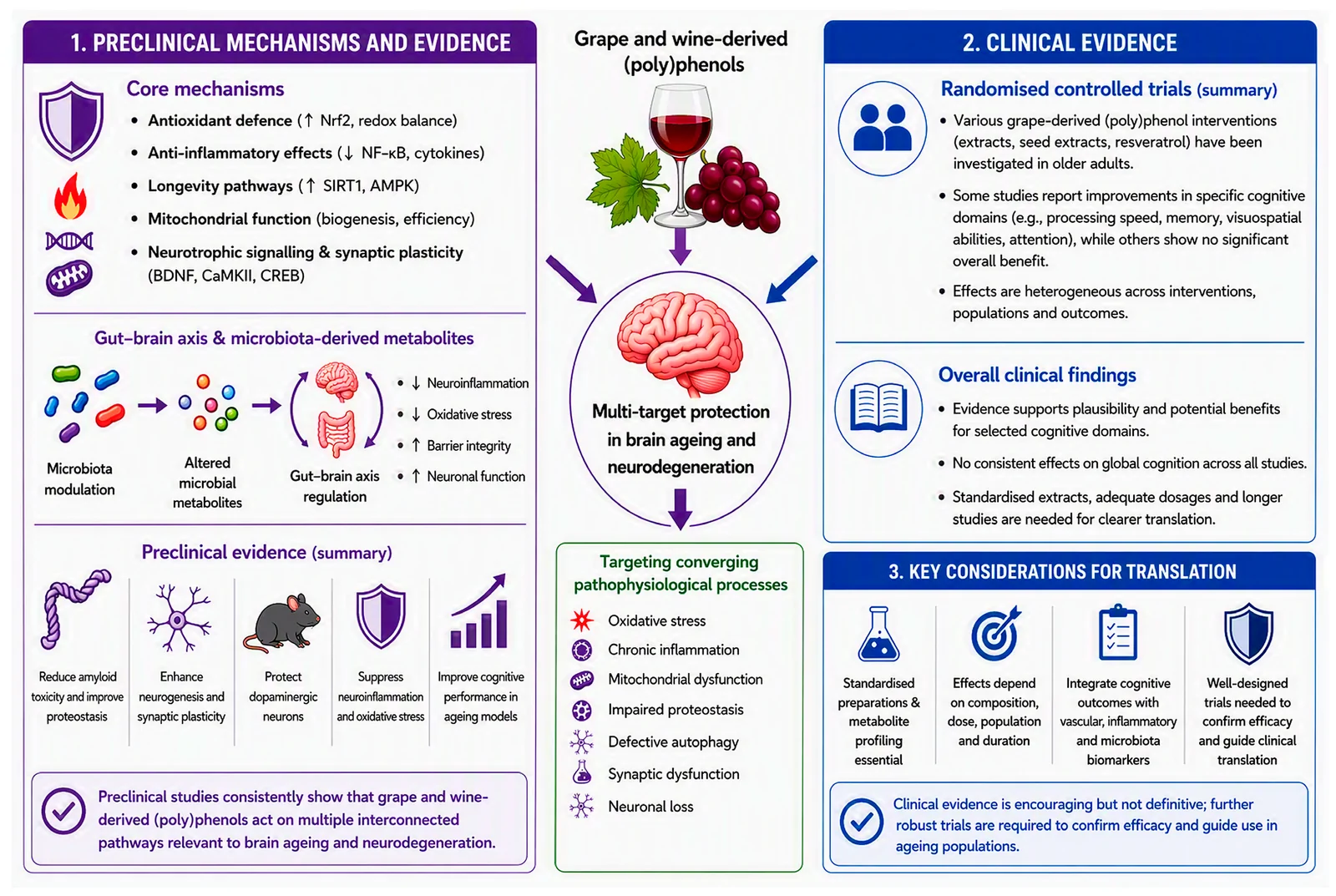
Summary of evidence linking grape- and wine-derived (poly)phenols to brain ageing and cognitive health. Grape- and wine-derived (poly)phenols influence multiple biological pathways implicated in brain ageing, including oxidative stress, inflammation, mitochondrial dysfunction, proteostasis, autophagy, neurotrophic signalling, and synaptic plasticity. Preclinical evidence consistently supports neuroprotective effects across these mechanisms. Clinical studies show more variable outcomes, with potential benefits primarily in specific cognitive domains such as memory, attention, and processing speed. Overall, findings support biological plausibility but highlight the need for standardised interventions and improved translational study design.

**Table 1:** Human intervention studies of grape-derived preparations and isolated resveratrol relevant to cognitive ageing and brain health.

Intervention Type	Study and Population	n	Dose and Duration	Comparator	Outcomes	Findings and Interpretation	Reference
Mixed grape-blueberry extract	Adults aged 60–80 years with mild cognitive impairment (MCI)	143	Memophenol^®^, 150 mg twice daily; 6 months	Placebo	Processing speed, visuospatial learning, BRIEF-A and other cognitive/self-report measures	Greater improvements in selected processing-speed, visuospatial-learning and BRIEF-A outcomes. Effects were not uniform across measures.	[89]
Grape seed procyanidin extract (GSPE)	Adults aged 60–85 years with mild cognitive impairment (MCI)	71	320 mg/day; 6 months	Placebo	Montreal Cognitive Assessment (MoCA)	Scores increased in both groups, but the between-group change was not significant.	[91]
Standardised grape extract	Cognitively healthy adults aged > 55 years	96	Cognigrape^®^, 250 mg/day; 12 weeks	Placebo	MMSE, RBANS, ENB-2 and Modified Bells Test	Improvements were reported across several cognitive domains, including memory, visuospatial ability, language and attention; findings are preparation-specific.	[90]
Isolated resveratrol	Adults aged 50–75 years with overweigh	46	200 mg/day; 26 weeks	Placebo	Memory, hippocampal functional connectivity and glucose-related measures	Improved word retention and increased hippocampal functional connectivity.	[12]
Isolated resveratrol	Adults aged >49 years with mild to moderate Alzheimer’s disease	119	500 mg orally once daily (with dose escalation by 500 mg increments every 13 weeks, ending with 1000 mg twice daily); 52 weeks	Placebo	Safety, plasma/CSF exposure, biomarkers and clinical outcomes	Resveratrol and metabolites were measurable in plasma and CSF; biomarker changes were reported, but their clinical significance remained uncertain.	[92]
Isolated resveratrol	Adults aged 35–60 years with overweight or obesity	99	500 mg/day; 12 weeks	Placebo	Acute/chronic cognition, gut microbiota, urinary metabolome, adiposity, inflammatory and oxidative markers	Baseline cognitive differences complicated interpretation. No significant within- or between-group differences were observed for microbiome, urinary metabolome, adiposity, inflammatory or oxidative markers.	[93]

Note: No eligible cognition trials involving alcoholic wine or dealcoholised wine were identified for inclusion in this clinical evidence table. The interventions shown comprise grape-derived extracts, a mixed grape-blueberry preparation and isolated resveratrol. These should not be interpreted as equivalent to one another or to wine consumption.

## Data Availability

No new data were created or analysed in this study. Data sharing is not applicable to this article.
